# The Binding Mechanism Between Inositol Phosphate (InsP) and the Jasmonate Receptor Complex: A Computational Study

**DOI:** 10.3389/fpls.2018.00963

**Published:** 2018-07-18

**Authors:** Mengqi Cui, Juan Du, XiaoJun Yao

**Affiliations:** ^1^Shandong Province Key Laboratory of Applied Mycology, College of Life Science, Qingdao Agricultural University, Qingdao, China; ^2^College of Chemistry and Chemical Engineering, Lanzhou University, Lanzhou, China

**Keywords:** jasmonate receptor, Inositol phosphate (InsP), binding mechanism, molecular docking, molecular dynamics simulation

## Abstract

Jasmonates are critical plant hormones, mediating stress response in plants and regulating plant growth and development. The jasmonate receptor is a multi-component complex, composed of Arabidopsis SKP-LIKE PROTEIN1 (ASK1), CORONATINE INSENSITIVE 1 (COI1), inositol phosphate (InsP), and jasmonate ZIM-domain protein (JAZ). COI1 acts as multi-component signaling hub that binds with each component. InsP is suggested to play important roles in the hormone perception. How InsP binds with COI1 and the structural changes in COI1 upon binding with InsP, JA-Ile, and JAZ are not well understood. In this study, we integrated multiple computational methods, such as molecular docking, molecular dynamics simulations, residue interaction network analysis and binding free energy calculation, to explore the effect of InsP on the dynamic behavior of COI1 and the recognition mechanism of each component of the jasmonate receptor complex. We found that upon binding with InsP, JA-Ile, and JAZ1, the structure of COI1 becomes more compact. The binding of InsP with COI1 stabilizes the conformation of COI1 and promotes the binding between JA-Ile or JAZ1 and COI1. Analysis of the network parameters led to the identification of some hub nodes in this network, including Met88, His118, Arg120, Arg121, Arg346, Tyr382, Arg409, Trp467, and Lys492. The structural and dynamic details will be helpful for understanding the recognition mechanism of each component and the discovery and design of novel jasmonate signaling pathway modulators.

## Introduction

Jasmonates, a family of plant hormones, regulate a variety of plant physiology processes, such as plant development, wound response, as well as defense response to insect herbivory and pathogens (Browse, 2008). It is suggested that CORONATINE INSENSITIVE 1 (COI1) is the receptor of jasmonates (Katsir et al., 2008; Fonseca et al., 2009; Yan et al., 2009). Subsequently, Sheard et al. determined the structure of the *Arabidopsis* jasmonate receptor (Sheard et al., 2010), a multiple component complex of Arabidopsis SKP-LIKE PROTEIN1 (ASK1), COI1, and Jasmonate ZIM-domain protein 1 (JAZ1). COI1 is the F-box component of a Skp1/Cullin/F-box protein (SCF), which is an E3 ubiquitin ligase complex.

**Figure 1:**
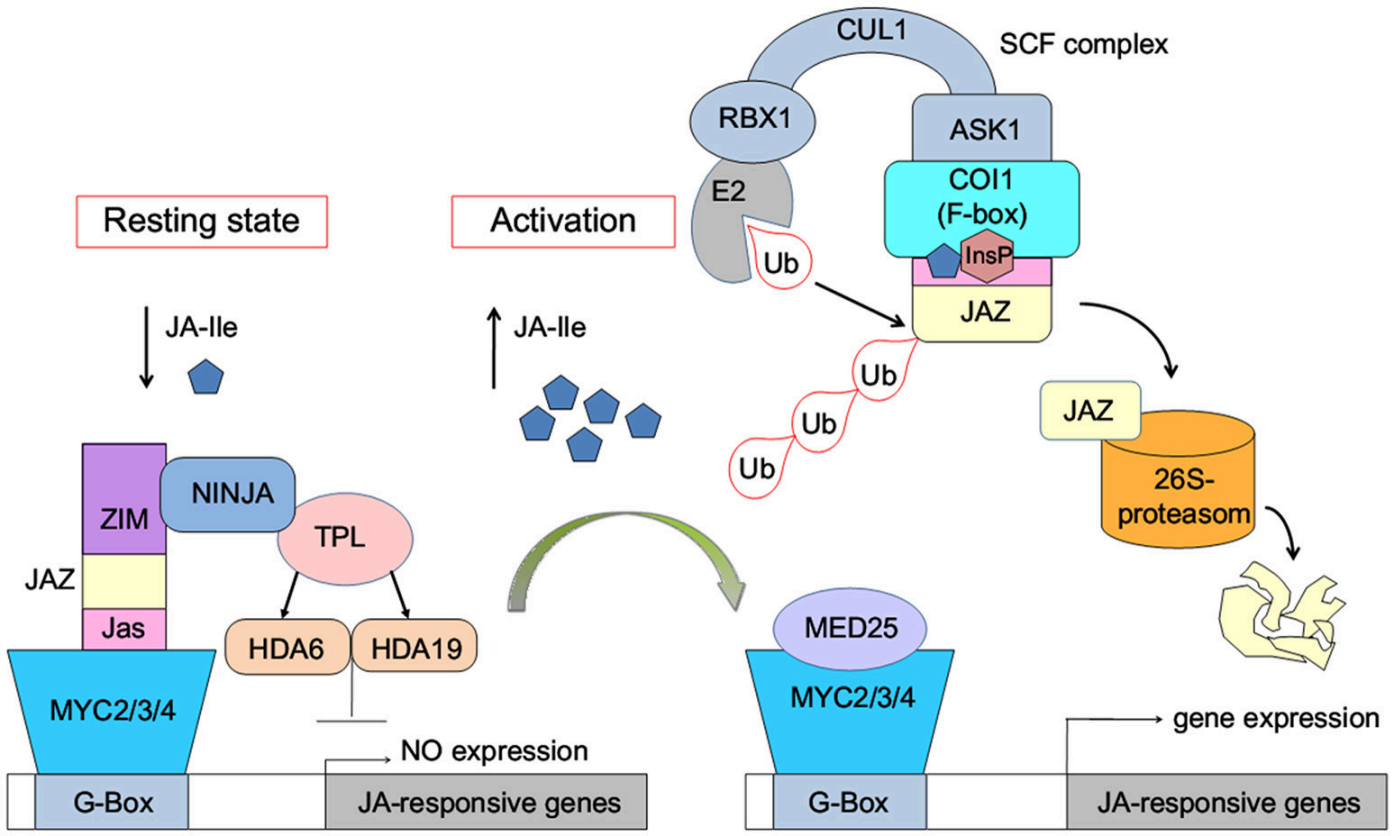
Model of JA-Ile perception via the SCF^COI1^-JAZ receptor complex (Wasternack and Hause, 2013; Wasternack and Strnad, 2015; Wasternack and Song, 2016). MYC2/3/4, binding to the G-box of a JA-Ile-responsive gene, is repressed by negative regulators such as jasmonate ZIM-domain proteins (JAZs). Novel Interactor of JAZ1 (NINJA) and TOPLESS (TPL), which act via HISTONE DEACETYLASE6 (HDA6) and HDA19, act as co-repressors. Ub, ubiquitin. InsP, inositol phosphate. E2, RBX1, CULLIN1 (CUL1), ASK1, and the F-box protein COI1 are components of the SCF-complex. MED25, subunit 25 of the Mediator complex.

The JA-induced gene expression process can be briefly described as follows: in the resting state, with low concentration of bioactive jasmonic acid (JA) conjugate JA-Ile, JAZ binds with MYC2/3/4 transcription factors to repress the expression of jasmonate-responsive genes (Figure 1). The endogenous JA-Ile concentration increases upon environmental or developmental stimulation. COI1 binds with JA-Ile and recruits JAZ transcriptional repressors, which leads to ubiquitination and proteasomal degradation of the JAZ repressors (Chini et al., 2007; Thines et al., 2007; Katsir et al., 2008; Sheard et al., 2010). MYC or other transcription factors are released after the degradation of JAZ. Subsequently, the expression of jasmonate-responsive genes was initialized (Chini et al., 2007).

**Figure 2:**
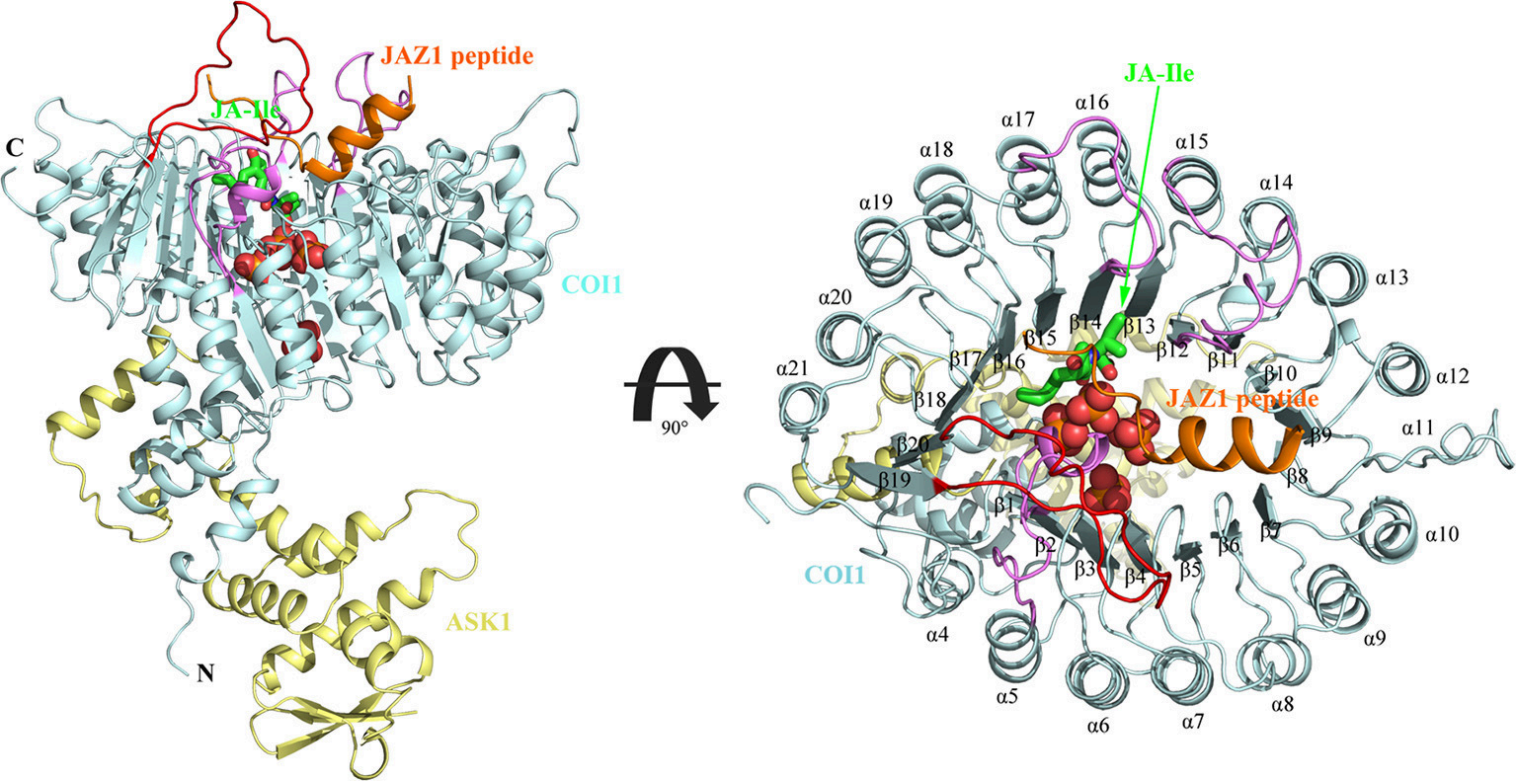
The overall structure of the ASK1-COI1 complex with JA-Ile, inorganic phosphate molecules, and the JAZ1 degron peptide (Sheard et al., 2010). ASK1-COI1 (yellow and cyan ribbons, respectively) with the JAZ1 degron peptide (orange ribbons), inorganic phosphate molecules are depicted as spheres (orange for phosphorus atoms and red for oxygen atoms) and JA-Ile in sticks (green for carbon atoms). Loops 2, 12, and 14 are colored in pink and loop C is colored in red.

There exists two domains of COI1, the N-terminal tri-helical F-Box domain, binding with ASK1, and a large C-terminal domain containing leucine-rich repeats (LRR) (Figure 2) (Xie et al., 1998; Sheard et al., 2010). This domain contains 18 tandem LRRs that form a horseshoe-shaped solenoid. Three long loops, the β2-α5 loop, β12-α15 loop, and β14-α17 loop at the top surface of this domain are involved in hormone and JAZ1 binding. JA-Ile binds into the solenoid of COI1. JAZ1 binds at the top surface of the solenoid. Sheard et al. also found that inositol pentakisphosphate (InsP_5_) binds to the bottom of COI1 solenoid and is involved in plant hormone perception (Sheard et al., 2010).

InsP_5_[3-OH] was purified together with the ASK1-COI1 expressed in insect cells by mass spectrometry and NMR analyses (Sheard et al., 2010). It also suggests Ins(1,4,5,6)P_4_, InsP_5_, InsP_6_ can promote jasmonate receptor assembly based on reconstitution assays, while it remains unknown which one is the physiologically relevant form. InsP_5_[3-OH] was displaced by phosphate molecules in the crystallization process. How it binds with COI1 remains unclear. A subsequent study suggested that InsP_5_ is involved in the perception of JA-Ile (Mosblech et al., 2011). Recently, Laha et al. found that inositol pyrophosphate InsP_7_ and InsP_8_ also promotes the binding between COI1 and JAZ1, which exert important functions in regulation of jasmonate-dependent responses (Laha et al., 2015). In addition, they found that JA treatment leads to InsP_8_ accumulation in plant, but not InsP_5_. Subsequently, the binding specificity of different inositol phosphates (InsPs) with ASK1-COI1 was explored by *in vitro* reconstitution experiments and molecular docking (Laha et al., 2016). The predicted binding mode suggests that InsP_8_ can form more favorable interaction with COI1 than InsP_5_, which explains that InsP_5_ acts as a weaker co-factor than InsP_8_ *in vivo* (in yeast). While the dynamics behavior of COI1 responding to InsP_8_, JA-Ile, and JAZ1 and the recognition mechanism of each component of jasmonate receptor are not well-understood.

In this study, the interaction mechanism between InsPs and the jasmonate receptor complex was investigated by multiple computational methods, such as molecular docking, molecular dynamics simulation, and binding free energy calculation. The recognition of each component of the complex was investigated by residue interaction network analysis. Our results provide information on the molecular recognition of InsP_8_ by COI1 and describe how InsP_8_ modulate the conformational dynamics of COI1. The detailed interaction mechanism of each component of the jasmonate receptor was also illustrated. This information will be helpful for the understanding of the recognition mechanism of each component of the jasmonate receptor complex. The results will facilitate the discovery and design of novel jasmonate signaling pathway modulators.

## Materials and methods

### Structure preparation

The X-ray crystal structure of the jasmonate receptor (PDB ID: 3OGM) was downloaded as the initial structure. The missing regions in this structure were built by Rosetta (De et al., 2015) and Modeller (Eswar et al., 2016), which comprises residue numbers 68–79 in ASK1 and 550–562 (loop C) in COI1 (68-SKAEAVEGAATS-79, 550-VPEVNQQGEIREM-565). This structure was then refined *in vacuo* by using Amber 12 (Pearlman et al., 1995). AMBER ff03 force field was assigned to the protein (Wang et al., 2004; Hornak et al., 2006). In the first stage, the side chains were relaxed by restraining the backbone atoms of the protein (5,000 cycles of steepest descent and 2,000 cycles of conjugate gradient minimizations); second, the whole protein were relaxed without any restrains (5,000 cycles of steepest descent and 2,000 cycles of conjugate gradient minimizations).

The structures of inositol phosphates used for docking were extracted from the protein structures in the Protein Data Bank (PDB). The structures of InsP_5_[3-OH], InsP_6_, and 1,5-InsP_8_ were extracted from the protein coded by 1FHW (Ferguson et al., 2012), 2P1P (Tan et al., 2007), and 3T9F (Wang et al., 2011), respectively. The other isoforms of InsP_5_ and InsP_8_ were sketched by Accelrys Discovery Studio 2.5 2010. The subsequent energy minimization of InsPs and JA-Ile was performed at the HF/6-31G^*^ level of Gaussian09 program (Frisch et al., 2009).

### Molecular docking

AutoDock Vina program (Trott and Olson, 2010) was used to dock all the forms of InsPs into the binding pocket of COI1. Autodock tools (ADT) (Morris et al., 2009) was employed to prepare the protein and InsPs. All water molecules were deleted, polar hydrogens were added and Gasteiger partial charges were assigned for the protein. The torsions were determined for InsPs. The protein was kept rigid. The grid center was determined according to the center of four phosphate molecules, with searching space size of 15 Å. The global search exhaustiveness value was set to 50. The maximum energy difference between the optimal binding mode and the worst-case was set to 5 kcal/mol. The structures were visualized using PyMOL (Schrödinger LLC., 2010).

### MD simulation

In order to explore the underlying dynamics simulation and interaction mechanism of jasmonate receptor and 1,5-InsP_8_, JA-Ile, and JAZ1, we set up six systems. The composition, water molecules and total atoms of all the systems are included in Table 1. MD simulations were performed for each system by NAMD2.9 (Phillips et al., 2005) with CHARMM27 force field (Mackerell et al, 2004) for the protein. The force field parameters for the 1,5-InsP_8_ and JA-Ile were built by the SwissParam service (Zoete et al., 2011). The protonation state of the ionized residue at PH 8 was determined on the basis of the predicted pKa value by the H++ server (Gordon et al., 2005). An explicit water solvent box with TIP3P waters was used to systems, and 0.15 M NaCl were added to neutralize electric charge of the box. For each system, 50,000 time steps of energy minimization were conducted with a harmonic force constraint on the ligand (InsP8 and JA-Ile) and protein, ligand and backbone of protein and ligand and Cα atoms of protein, respectively. After that the temperature was raised from 0 to 300 K gradually within 200 ps under the NVT ensemble. After 2 ns equilibration under the NPT ensemble condition, 100 ns MD production was performed. The SHAKE algorithm was applied to restrain the bond length relating with hydrogen atoms (Coleman et al., 1977) and Particle Mesh Ewald (PME) (Tom et al., 1993) method was used to calculate the long electrostatic interactions. A 12.0 Å cut-off was set to calculate the van der Waals interactions. The integration step was set to 2 fs. The coordinates were preserved every 10 ps for trajectory analysis (Humphrey et al., 1996). The MD simulation data has been submitted to the zendo database (https://zenodo.org/) with accession links: https://zenodo.org/record/1255820.

**Table 1:** Summary of the six simulation systems.

**System**	**System composition**	**Water molecules**	**Total atoms**	**Time (ns)**
1	COI1, ASK1	37,488	124,338	100
2	COI1, ASK1, 1,5-InsP_8_	37,462	124,324	100
3	COI1, ASK1, 1,5-InsP_8_, JA-Ile	37,468	124,394	100
4	COI1, ASK1, JAZ1, JA-Ile	37,373	124,370	100
5	COI1, ASK1, JAZ1, 1,5-InsP_8_	37,355	124,382	100
6	COI1, ASK1, JAZ1, 1,5-InsP_8_, JA-Ile	37,347	124,356	100

### Residue interaction network analysis

For each system, the structures extracted from the final 20-ns MD simulation trajectory, were submitted to Residue Interaction Network Generator 2.0 (RING 2.0) server (Martin et al., 2011; Piovesan et al., 2016) for residue interaction network (RIN) analysis. The network was visualized by the plugin RINalyzer (Doncheva et al., 2011) integrated in Cytoscape (Shannon et al., 2003). The nodes denote amino acid residues or ligands and the edges represent residue-residue interactions, which include the interaction between the closest atoms, hydrogen bonding, salt bridge, ionic interaction, π-π stacking, and van der Waals interaction.

In addition, to characterize the residue interaction network of COI1, the parameters including shortest path betweenness and closeness centrality were calculated by the NetworkAnalyzer (Assenov et al., 2008) plugin of Cytoscape. The value of them is between 0 and 1 (Doncheva et al., 2012). It is suggested that residues with high shortest path betweenness values play an important role in stabilizing the protein structures, as well as those with high closeness values are likely significant for the protein function (Vendruscolo et al., 2002; Amitai et al., 2004; Brinda and Vishveshwara, 2005). Residue-residue communication is critical for the function of a protein (Süel et al., 2003). The closeness centrality measures how fast the information flows from a node to other reachable nodes in a network (Freeman, 1978).

### Binding free energy calculation

The binding free energy of JA-Ile or JAZ1 with COI1 was calculated according to Molecular Mechanics/Generalized Born Surface Area (MM-GBSA) method using Prime/MM-GBSA module (Prime) of Schrödinger (Schrödinger, LLC, New York, NY)(2005). The total of 100 COI1—JA-Ile and COI1—JAZ1 complexes was extracted from the equilibrated MD trajectories. The complexes were minimized and the energies of the complex were calculated using the OPLS_2005 force field and VSGB solvation model (Jianing et al., 2011). The binding free energy is estimated according to the following equation:

(1) $$\begin{array}{r} \Delta {\text{G}}_{\text{bind}}={\text{G}}_{\text{complex}}-{\text{G}}_{\text{receptor}}-{\text{G}}_{\text{ligand}} \end{array}$$

(2) $$\begin{array}{r} \Delta {\text{G}}_{\text{bind}}=\Delta {\text{E}}_{\text{MM}}+\Delta {\text{G}}_{\text{GB}}+\Delta {\text{G}}_{\text{SA}} \end{array}$$

Where G_complex_ is the minimized free energy for the complex, G_receptor_ and G_ligand_ are the minimized free energy for the free COI1 and free ligand (JA-Ile or JAZ1). Each energy term was calculated by a summation of molecular mechanics energy (ΔE_MM_), GBSA solvation energy (ΔG_GB_), and surface area energy (ΔG_SA_). Strain energy was calculated for both COI1 and JA-Ile or JAZ1.

Statistical analysis was performed to affirm the significance of the difference of binding free energy. A one-way ANOVA was conducted here to test for significant differences (*P* < 0.05) between system 3/4 (COI1+ASK1+1,5-InsP_8_+JA-Ile/ COI1+ASK1+JAZ1+JA-Ile) and system 6 for the binding free energy of COI1—JA- Ile, and system 4/5 (COI1+ASK1 +JAZ1 +JA-Ile/ COI1+ASK1+ JAZ1+1,5-InsP_8_) and system 6 (COI1+ASK1+JAZ1+1,5-InsP_8_+JA-Ile) for the binding free energy of COI1—JAZ1.

## Results

### The binding mode between InsPs and the jasmonate receptor complex

The structure of Loop C from COI1 and missing regions ASK1 were built by Rosetta and Modeler, which is shown in Figure 2. The built model was subsequently minimized by NAMD. NAMD is one of the most efficient open-source program for molecular dynamics simulation, which is widely applied to explore the dynamic behavior of large systems (millions of atoms). To compare the binding affinity between COI1 and different forms of InsPs, InsP_5_s, InsP_6_, and InsP_8_s were all docked to the binding site of phosphate ions in the refined full structure of jasmonate receptor complex. Autodock vina is an efficient and accurate docking program widely used in predicting the binding mode and binding affinity between protein and ligand (Trott and Olson, 2010; Wang et al., 2016). The vina score of each InsP is summarized in Table 2. It shows that InsP_8_s exhibits lower binding free energy than InsP_5_s and InsP_6_.

**Table 2:** Vina score for different forms of InsPs.

**Receptor**	**Ligand**	**Vina score (kcal/mol)**
COI1+ASK1+JAZ1+JA-Ile	1,2-InsP_8_	−7.1
	1,3-InsP_8_	−6.7
	1,4-InsP_8_	−6.8
	1,5-InsP_8_	−7.0
	1,6-InsP_8_	−6.9
	InsP_6_	−6.4
	InsP_5_[1-OH]	−6.4
	InsP_5_[2-OH]	−6.3
	InsP_5_[3-OH]	−6.5
	InsP_5_[4-OH]	−6.3
	InsP_5_[5-OH]	−6.2
	InsP_5_[6-OH]	−6.4

The detailed binding mode of each form of InsPs with the lowest binding free energy is also shown in Figure S1. InsP_5_ forms hydrogen bond interaction with Lys79, Lys81, Arg85, Arg120, Lys147, Arg409, and Arg440 from COI1 and Arg206 from JAZ1 (Figure S1A). It also interacts with Trp467 of COI1 through hydrophobic interaction. Regarding InsP_6_, it forms hydrogen bond interaction with Lys79, Lys81, Arg85, Arg120, Arg121, Arg346, Arg409, and Arg440 from COI1 and Arg206 from JAZ1 and hydrophobic interaction with Met88 and Trp467 from COI1. For InsP_8_s, 1,2-InsP_8_ shows the lowest binding free energy (−7.1 kcal/mol) with COI1 in all the isoforms. The second-lowest binding free energy is for 1,5-InsP_8_, with the score of −7.0 kcal/mol. Comparison of the detailed binding mode of these two isoforms, shows that 1,2-InsP_8_ forms hydrogen bond interaction with Lys79, Lys81, Arg85, Arg120, Arg346, Arg409, and Arg440 from COI1 and Arg206 from JAZ1 and hydrophobic interaction with Trp467 of COI1. 1,5-InsP_8_ forms hydrogen bond interaction with Lys79, Lys81, Arg85, His118, Arg120, Arg121, Lys144, Lys147, Arg346, Arg409, and Lys492 from COI1 and Arg206 from JAZ1 and hydrophobic interaction with Met88 of COI1. A previous study shows that His118, Arg346, and Lys492 from COI1 are important for the binding of InsP_8_ with COI1 (Laha et al., 2015); and 1,5-InsP_8_ can form hydrogen bond interaction with all of these residues. However, 1,2-InsP_8_ does not interact with His118 and Lys492. Based on the predicted binding free energy and binding mode, 1,5-InsP_8_ was selected for the further MD simulations.

### Structure and dynamics behavior of jasmonate receptor

**Figure 3:**
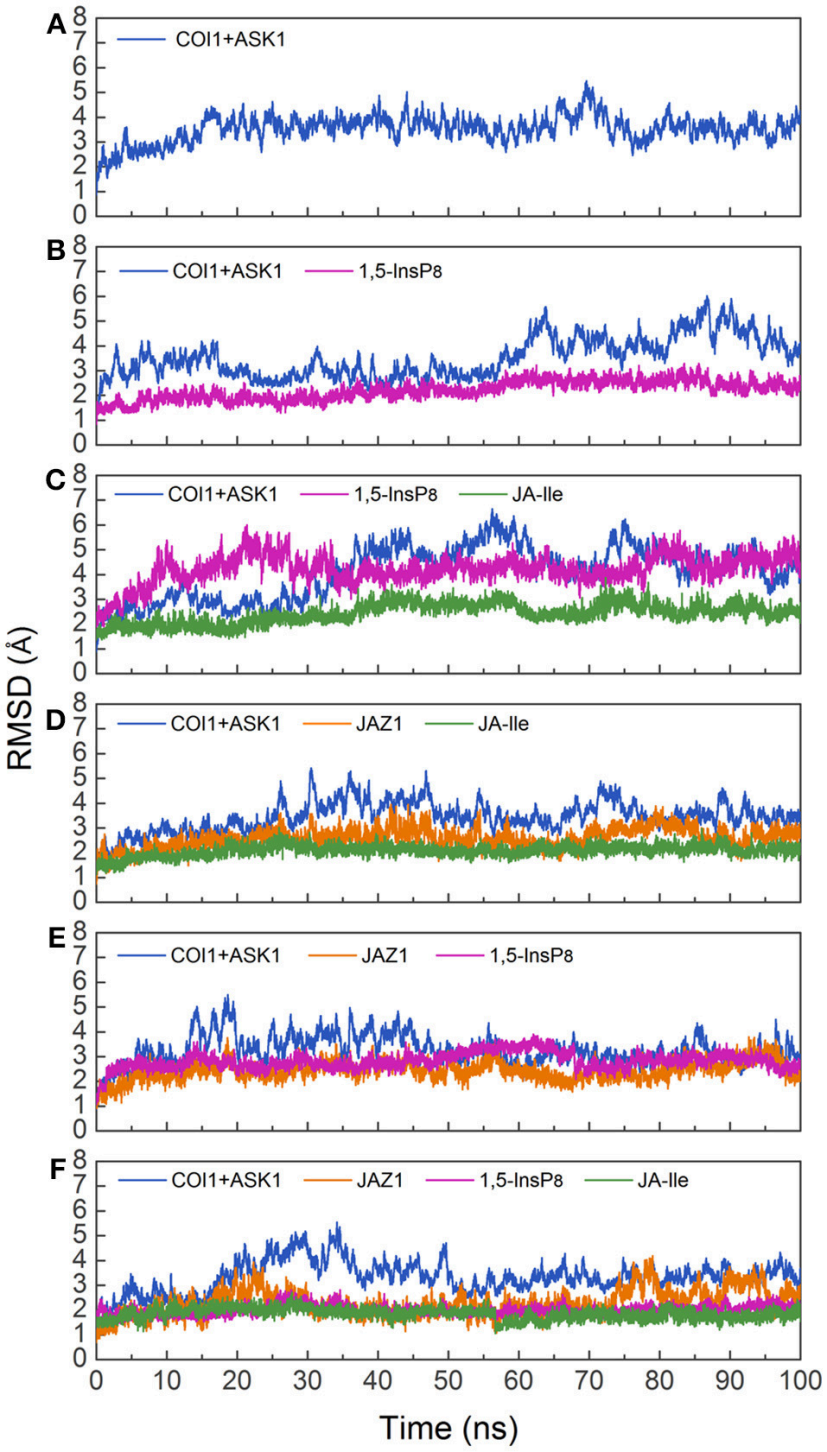
RMSD plots of the complex during molecular dynamics simulation with respect to the crystal structure. **(A)** system 1, COI1+ASK1; **(B)** system 2, COI1+ASK1+1,5-InsP_8_; **(C)** system 3, COI1+ASK1+1,5-InsP_8_+JA-Ile; **(D)** system 4, COI1+ASK1+JAZ1 +JA-Ile; **(E)** system 5, COI1 +ASK1 +JAZ1+1, 5-InsP_8_; **(F)** system 6, COI1+ ASK1+JAZ1 +1,5-InsP_8_+JA-Ile.

To investigate the stability and conformational differences of the jasmonate receptor in response to the binding with InsP_8_, JA-Ile, and JAZ1, we carried out molecular dynamics simulations of the jasmonate receptor structures in six systems. The conformational stability of six systems during MD simulations was evaluated by calculating the root mean square deviation (RMSD) of the backbone atoms. Figure 3 reflected the trend of RMSD in the six systems relative to the crystal structure.

Since there was no InsP molecule in the structure of jasmonate receptor (PDB ID: 3OGM), the complex was constructed based on the docked pose with the lowest vina score of 1,5-InsP_8_. MD simulations were constructed to examine the stability and the effect of 1,5-InsP_8_, JA-Ile, and JAZ1 with the jasmonate receptor. The six systems (systems 1–6) achieved equilibrium at 20, 65, 55, 30, 20, and 35 ns, respectively. The RMSD values of the backbone atoms converged at 3.27–4.68 Å in six systems. The average RMSD values of 1,5-InsP_8_ are 2.52, 4.38, 2.87, and 2.00 Å in system 2 (COI1+ASK1+1,5-InsP_8_), system 3 (COI1+ASK1+1,5-InsP_8_+JA-Ile), system 5 (COI1+ASK1+JAZ1+1,5-InsP_8_), and system 6 (COI1+ASK1+JAZ1+1,5-InsP_8_+JA-Ile), respectively. For JA-Ile, the average RMSD values are 2.59, 2.13, and 1.79 Å in system 3, system 4 (COI1+ASK1+JAZ1+JA-Ile), and system 6, respectively. In addition, the averaged RMSD of JAZ1 is 2.67, 2.49, and 2.33 in the system 4, system 5, and system 6, respectively. Thus, based on the RMSD results, our MD simulations are reliable for further investigation.

**Figure 4:**
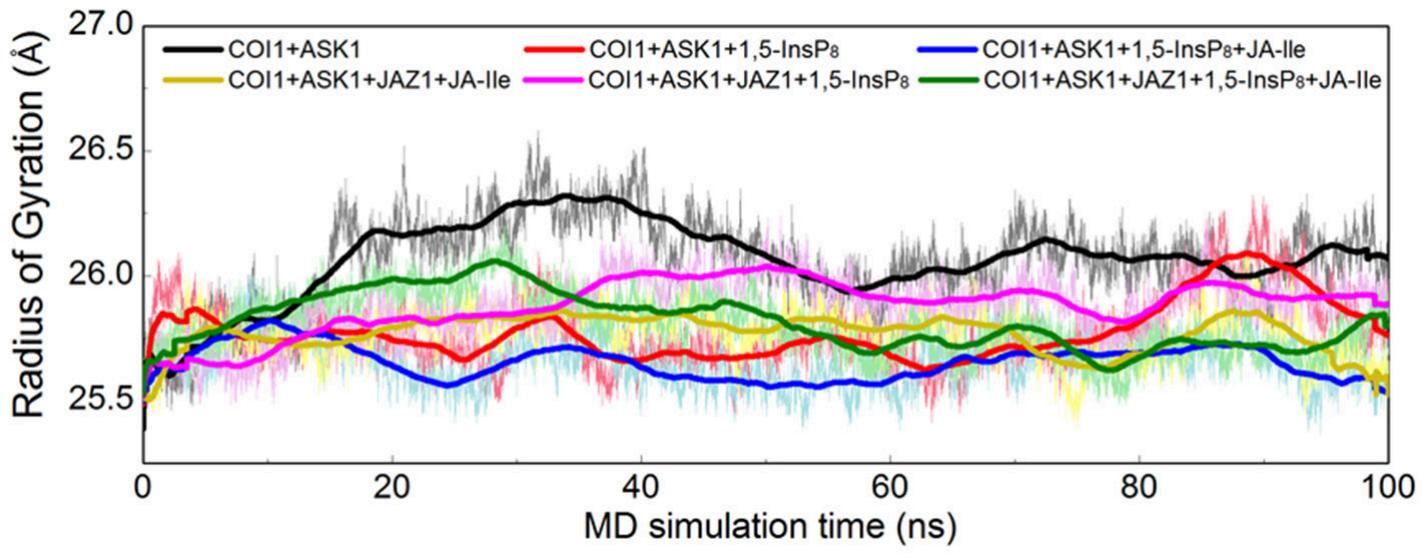
Rg plots of backbone atoms of protein for the six systems. Black is used for system 1 (COI1+ASK1), red for system 2 (COI1+ASK1+1,5-InsP_8_), blue for system 3 (COI1+ASK1+1,5-InsP_8_+JA-Ile), yellow for system 4 (COI1+ASK1+JAZ1+JA-Ile), magenta for system 5 (COI1+ASK1+JAZ1+1,5-InsP_8_), and green for system 6 (COI1+ASK1+JAZ1+1,5-InsP_8_+JA-Ile).

Furthermore, the radius of gyration (Rg) of COI1 in six systems was also calculated to detect the compactness of its structure, as shown in Figure 4. We calculated the average Rg ranging from 40 to 100 ns in each system. The average Rg of COI1 is the highest in system 1 (COI1+ASK1), with the value of 26.03 ± 0.11 Å. The next is that in system 5 (COI1+ASK1+JAZ1+1,5-InsP_8_), with the value of 25.90 ± 0.09 Å. The COI1 shows average Rg values in system 2 (COI1+ASK1+1,5-InsP_8_) of 25.75 ± 0.16 Å and system 4 (COI1+ASK1+JAZ1+JA-Ile) of 25.73 ± 0.11 Å. The mean Rg values of COI1 was 25.60 ± 0.09 Å and 25.71 ± 0.09 Å in systems 3 (COI1+ASK1+1,5-InsP_8_+JA-Ile) and 6 (COI1+ASK1+JAZ1+1,5-InsP_8_+JA-Ile), respectively. Comparing with other systems, it indicates that the structure of COI1 in system 1 (COI1+ASK1) without any ligands, also termed as the *apo* system, was less compact.

**Figure 5:**
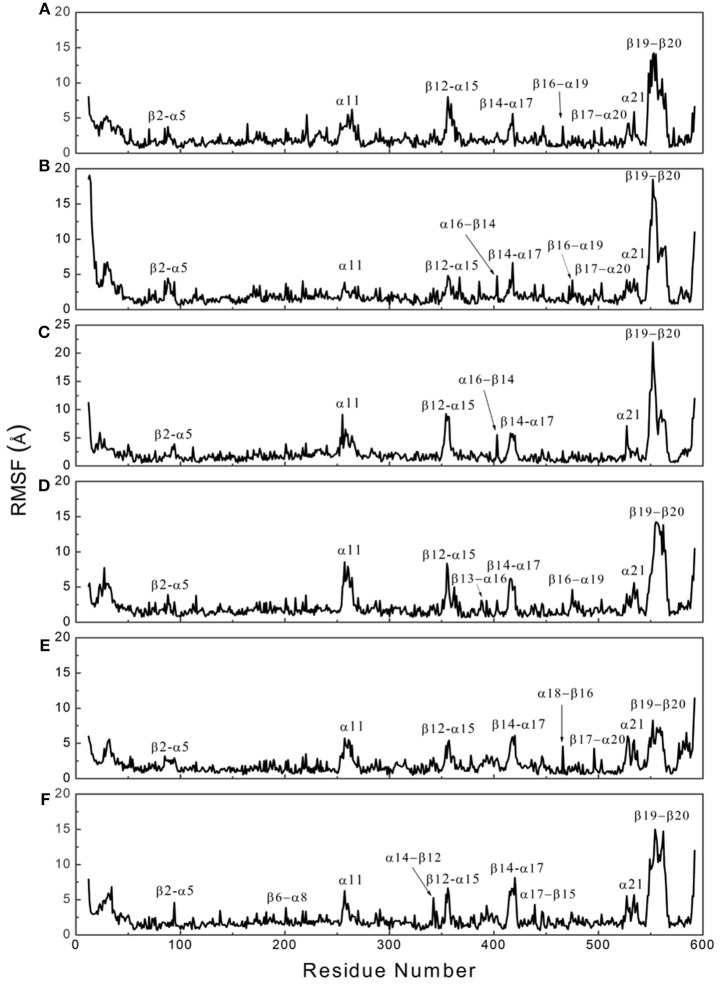
RMSF plots of Cα atoms of COI1 in the six systems. **(A)** system 1, COI1+ASK1; **(B)** system 2, COI1+ASK1 +1,5-InsP_8_; **(C)** system 3, COI1+ASK1+1,5-InsP_8_+JA-Ile; **(D)** system 4, COI1+ASK1+JAZ1 +JA-Ile; **(E)** system 5, COI1+ASK1+JAZ1+1,5-InsP_8_; **(F)** system 6, COI1+ASK1+JAZ1+1,5-InsP_8_+JA-Ile.

**Figure 6:**
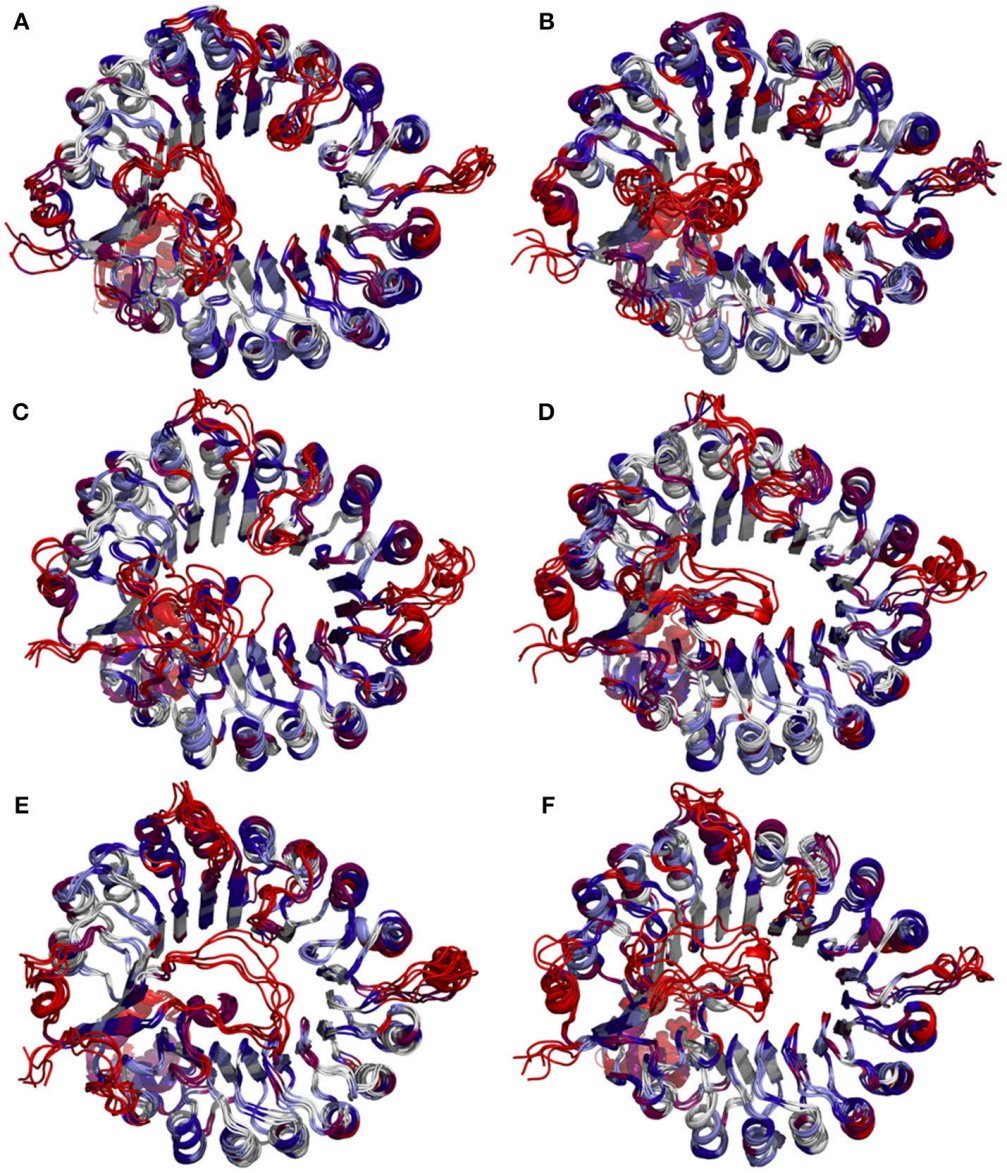
Snapshots of COI1 structures from MD trajectories. The color is coded by the RMSF value, with blue being the lowest fluctuations to light blue to white to magenta to red being the highest fluctuations. **(A)** system 1, COI1+ASK1; **(B)** system 2, COI1+ASK1 +1,5-InsP_8_; **(C)** system 3, COI1+ASK1+1,5-InsP_8_+JA-Ile; **(D)** system 4, COI1+ASK1+JAZ1 +JA-Ile; **(E)** system 5, COI1+ASK1+JAZ1+1,5-InsP_8_; **(F)** system 6, COI1+ASK1+JAZ1+1,5-InsP_8_+JA-Ile.

To determine whether binding with InsP_8_, JA-Ile, and JAZ1 affected the dynamic behavior of COI1 residues, the mobility of the protein residues was examined by plotting the root-mean-square fluctuation (RMSF) of the backbone atoms of COI1 versus the residue number (Figure 5). We can observe that most of high flexible regions are the loops linking the regular secondary elements or those on the surface of COI1, such as β2-α5 (termed as loop 2), β12-α15 (loop 12), β14-α17 (loop 14), β19-β20 loop (loop C), α11, and α21. The large fluctuations may be caused by the intrinsic flexibility of this protein, in the light of these regions exhibit high flexibility in all the systems. The main flexible regions were identified for all systems. The locations of these regions in the protein structure are shown in Figure 6. The β16-α19 loop and β17-α20 loop located at the top surface of the COI1 solenoid, exhibit high flexibility in system 1 (COI1+ASK1) and system 2 (COI1+ASK1+1,5-InsP_8_). The α16-β14 loop located close to 1,5-InsP_8_ also shows high flexibility in system 2 and system 3 (COI1+ASK1+1,5-InsP_8_+JA-Ile). The β13-α16 and β16-α19 loops, at the top of the COI1, exhibit high flexibility in system 4 (COI1+ASK1+JAZ1+JA-Ile). In system 5 (COI1+ASK1+JAZ1+1,5-InsP_8_), loop β17-α20 at the top of the COI1 shows high flexibility. In addition, the α18-β16 loop is close to 1,5-InsP_8_, exhibiting high flexibility. In system 6 (COI1+ASK1+JAZ1+1,5-InsP_8_+JA-Ile), the β6-α8 loop is located close to JAZ1, the α14-β12 loop and α17-β15 loop are close to the bottom of 1,5-InsP_8_, exhibiting high flexibility.

### The interaction mode between InsP_8_ and COI1 after MD simulations

**Figure 7:**
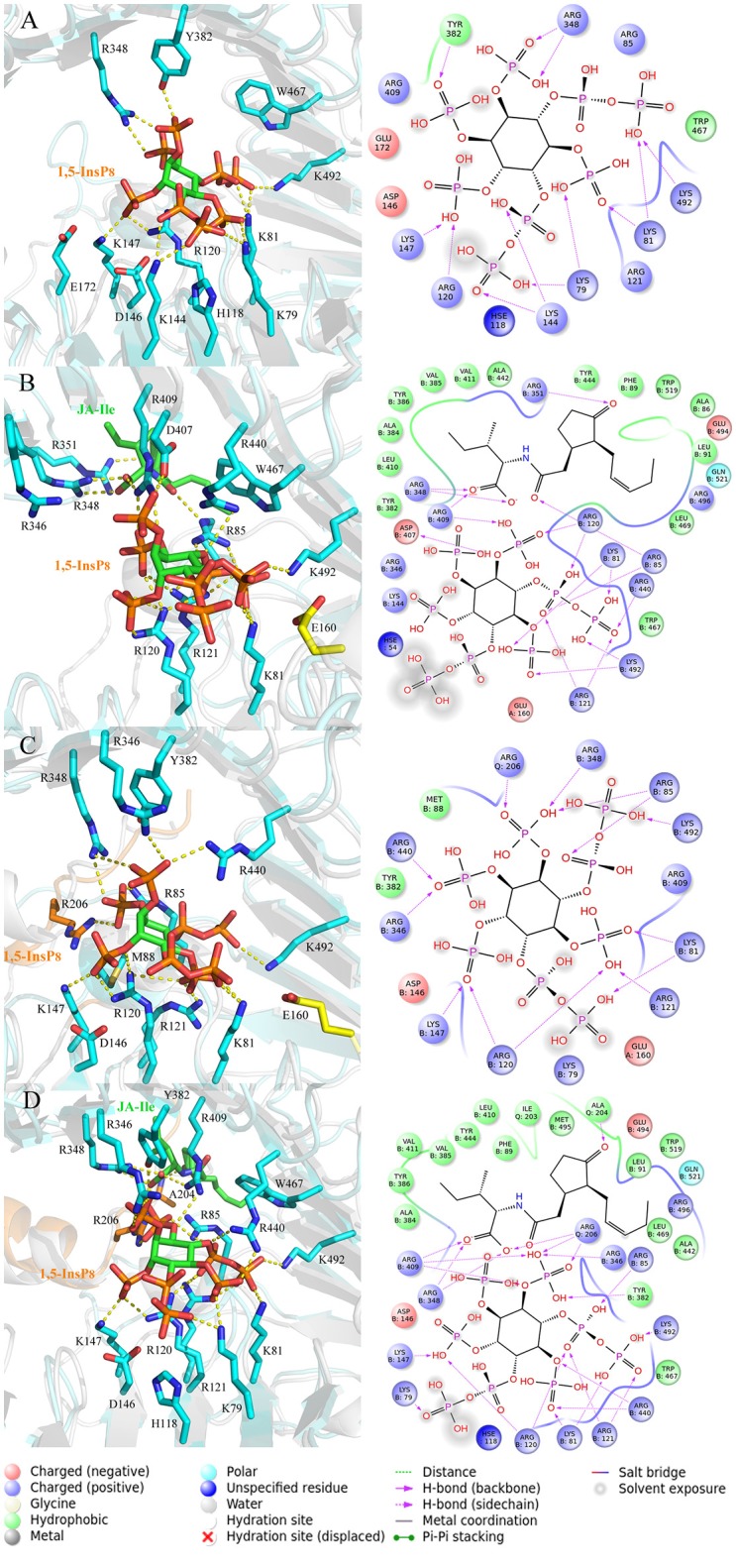
The representative snapshots from the last 20 ns of the MD trajectories. **(A)** system 2, COI1+ASK1 +1,5-InsP_8_; **(B)** system 3, COI1+ASK1+1,5-InsP_8_+JA-Ile; **(C)** system 5, COI1+ASK1+JAZ1+1,5-InsP_8_; **(D)** system 6, COI1+ASK1+JAZ1+1,5-InsP_8_+JA-Ile. For panels **(A–D)**, the proteins are in white and cyan for the docked and MD structures, respectively. Hydrogens are omitted for clarity.

In order to analyze the interaction mode between 1,5-InsP_8_ and COI1 after MD simulations, we extracted the representative structures from the final 20-ns MD trajectory (Figure 7). The detailed binding mode of 1,5-InsP_8_ with COI1 reveals that the components, JA-Ile and JAZ1, are also important for the whole interaction network in the solenoid of COI1. In system 2 (COI1+ASK1+1,5-InsP_8_), 1,5-InsP_8_ maintains the hydrogen bond interaction with Lys79, Lys81, Arg120, Lys144, Lys147, and Lys492 from COI1 (Figure 7A). Additionally, 1,5-InsP_8_ forms novel hydrogen bond interaction with Arg348, as well as hydrophobic interaction with Try382 and Trp467 of COI1. The hydrogen bond interaction between 1,5-InsP_8_ and His118 or Arg346 from COI1 disappeared, which were shown to be important for the binding of InsP_8_ with COI1. In system 3 (COI1+ASK1+1,5-InsP_8_+JA-Ile), its interaction with Lys81, Arg85, Arg120, Arg121, Arg409, Arg440, and Lys492 from COI1 was maintained (Figure 7B), but the hydrogen bond interaction with His118 and Arg346 from COI1 was lost. Additionally, 1,5-InsP_8_ forms hydrogen bond with Asp407 and hydrophobic interaction with Tyr382 and Trp467 of COI1. In system 5 (COI1+ASK1+JAZ1+1,5-InsP_8_), the interaction with Lys81, Arg85, Arg120, Arg121, Lys147, Arg346, Arg440, and Lys492 from COI1 was maintained, as well as with Arg206 from JAZ1 and the hydrophobic interaction with Met88 (Figure 7C). The hydrogen interaction with His118 and Arg409 from COI1 was lost. It forms hydrogen bond with Arg348 and hydrophobic interaction with Tyr382. In system 6 (COI1+ASK1+JAZ1+1,5-InsP_8_+JA-Ile), the hydrogen interaction with Lys79, Lys81, Arg85, Arg120, Arg121, Lys147, Arg346, Arg440, Arg409, and Lys492 from COI1 was maintained, as well as that with Arg206 from JAZ1 (Figure 7D). The hydrogen interaction with His118 from COI1 was lost and hydrogen bond interaction with Arg348 and hydrophobic interaction with Tyr382 and Trp467 of COI1 was formed.

### Residue interaction networks

Investigation and resolution of residue interaction network (RIN) is imperative for the understanding protein structure-function relationships (Amitai et al., 2004; Del et al., 2006; Vishveshwara et al., 2009). Recently, RIN analysis has been successfully applied to investigate mutation effects, protein folding, domain-domain communication, and catalytic activity (Dokholyan et al., 2002; Swintkruse, 2004; Del et al., 2006; Soundararajan et al., 2010; Boehr et al., 2013; Scaini et al., 2014; Biswas et al., 2017). Based on the interaction mode analysis, we found that 1,5-InsP_8_ lost some interactions and formed novel interactions with the jasmonate receptor complex in different systems. To comprehensively understand the differences between RINs in the solenoid of COI1 binding with different ligands, we explored the relationship between crucial residues of COI1 solenoid and the ligands, including InsP_8_, JA-Ile, and JAZ1. The residue interaction networks were generated based on the representative structures extracted from the final 20-ns of MD trajectory.

**Figure 8:**
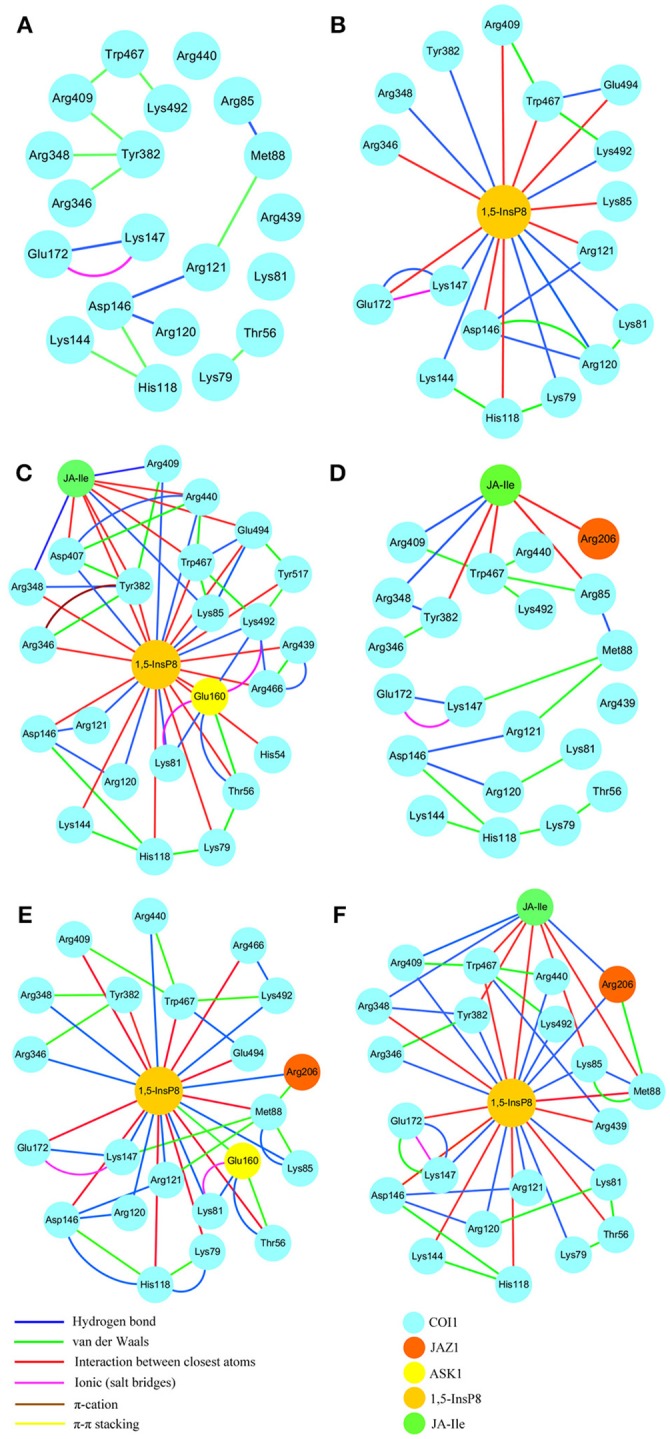
The residue interaction network of amino acids within 5Å around 1,5-InsP_8_. **(A)** system 1, COI1+ASK1, without 1,5-InsP_8_ and JA-Ile, the residue interaction network in the cavity of COI1; **(B)** system 2, COI1+ASK1+1,5-InsP_8_, the residue interaction network between 1,5-InsP_8_ and COI1; **(C)** system 3, COI1+ASK1+1,5-InsP_8_+JA-Ile, the residue interaction network among 1,5-InsP_8_, JA-Ile and COI1; **(D)** system 4, COI1+ASK1+JAZ1+JA-Ile, the residue interaction network in the cavity of COI1 and between JA-Ile and COI1; **(E)** system 5, COI1 +ASK1 +JAZ1 +1,5-InsP_8_, the residue interaction network between 1,5-InsP_8_ and COI1; **(F)** system 6, COI1+ASK1+JAZ1+1,5-InsP_8_+JA-Ile, the residue interaction network among 1,5-InsP_8_, JA-Ile, and COI1.

RIN plots shown in Figure 8 depicts the presence of interaction network among His118, Arg120, Arg121, Lys144, Asp146, and Met88 or Arg346, Arg348, Tyr382, Arg409, Trp467, and Lys492 in system 1 (COI1+ASK1, Figure 8A). The competent form of the *apo* structure exhibits the weakest network (system 1). Upon binding with 1,5-InsP_8_ (Figure 8B), 17 interactions are observed between 1,5-InsP_8_ and COI1, including 8 hydrogen bond interactions and 9 interactions between closest atoms. Upon addition of a new component, JA-Ile, the network became stronger in system 3 (COI1+ASK1+1,5-InsP_8_+JA-Ile); there are 24 interactions between 1,5-InsP_8_ and COI1, including 8 hydrogen bond interactions and 16 interactions between closest atoms (Figure 8C). In system 5 (COI1+ASK1+JAZ1+1,5-InsP_8_), 1,5-InsP_8_ forms 22 interactions with COI1, ASK1, and JAZ1, including 10 hydrogen bond interactions and 12 interactions between closest atoms (Figure 8E). In system 6 (COI1+ASK1+JAZ1+1,5-InsP_8_+JA-Ile), 1,5-InsP_8_ forms 22 interactions with COI1, ASK1, JA-Ile, and JAZ1, including 12 hydrogen bond interactions and 10 interactions between closest atoms (Figure 8F).

**Figure 9:**
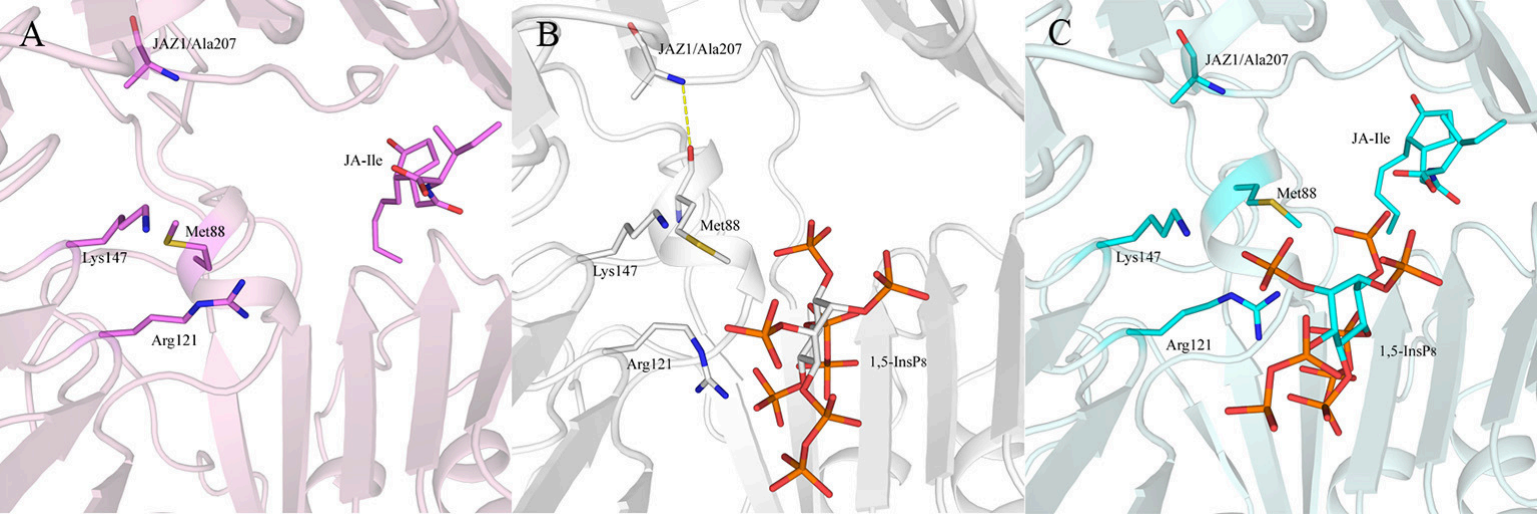
The interaction between 1,5-InsP_8_, JA-Ile, JAZ1 and COI1 (Met88, Arg121, Lys147) from the last 20 ns of the MD trajectories. **(A)** system 4 (COI1+ASK1+JAZ1+JA-Ile); **(B)** system 5 (COI1+ASK1+JAZ1+1,5-InsP_8_); **(C)** system 6 (COI1+ASK1+JAZ1+1,5-InsP_8_+JA-Ile). The carbon atoms of residues or ligands are colored by pink, white, and cyan in systems 4, 5, and 6, respectively. The COI1 and JAZ1 are colored by light pink, white, and palecyan in system 4, system 5, and system 6, respectively.

**Figure 10:**
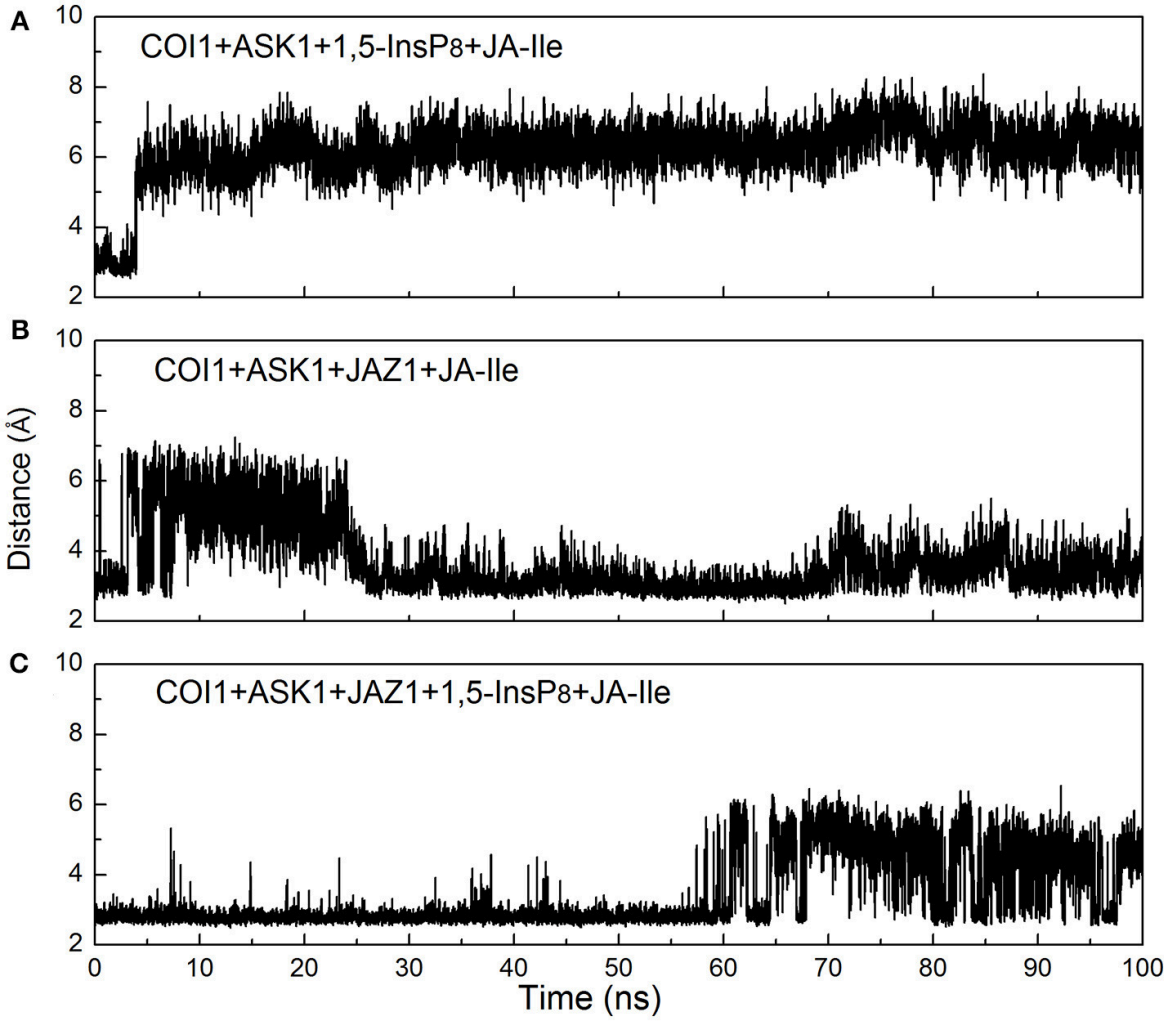
Monitoring of the intermolecular hydrogen bonds between JA-Ile and COI1 during the MD simulation, **(A)** system 3 (COI1+ASK1+1,5-InsP_8_+JA-Ile); **(B)** system 4 (COI1+ASK1+JAZ1+JA-Ile); **(C)** system 6 (COI1+ASK1+JAZ1+1,5-InsP_8_+JA-Ile).

Regarding systems 1 (COI1+ASK1) and 4 (COI1+ASK1+JAZ1+JA-Ile) that lack of 1,5-InsP_8_, the interaction network is obviously less stronger than that of other systems (Figures 8A,D). Compared with system 4 (Figure 8D), the van der Waals interaction between Met88 and Arg121 or Lys147 disappeared, which resulted in conformational variation of Met88 in system 6 (COI1+ASK1+JAZ1+1,5-InsP_8_+JA-Ile, Figure 8F). Met88 forms interaction of closest atom with JA-Ile and van der Waals interaction with JAZ1. Regarding system 5 (COI1+ASK1+JAZ1+1,5-InsP_8_), which lack JA-Ile, the conformation of 1,5-InsP_8_ changed greatly. Met88 forms hydrogen bond interaction with Ala207 of JAZ1 and van der Waals interaction with Arg121 or Lys147 (Figure 9). Hub nodes can clearly be found in the network inside COI1, including 1,5-InsP_8_, JA-Ile, Met88, His118, Arg120, Arg121, Arg346, Tyr382, Arg409, Trp467, and Lys492. The binding with 1,5-InsP_8_, JA-Ile, and JAZ1 significantly changes the network in COI1 solenoid. Most importantly, 1,5-InsP_8_ promotes and stabilizes the interaction of COI1—JA-Ile or COI1—JAZ1. The system lacking 1,5-InsP_8_ or JAZ1 also affects the hydrogen bond interaction of COI1—JA-Ile (Figure 10). The distance between Arg85 of COI1 and JA-Ile decreases in the presence of InsP_8_ or JAZ1.

To further compare the residue network changes in different systems, we calculated the two parameters of each node in the network: the shortest path betweenness and closeness centrality. Table 3 summarizes the value of critical residues shortest path betweenness and closeness centrality in the network in each system. It is found that 1,5-InsP_8_ possesses the highest value of betweenness in all the systems, suggesting that it is crucial in the COI1 network. The next highest value is found for JA-Ile. Both of them are crucial for stabilizing the conformation of COI1, which is important for the binding with JAZ1. In addition, the closeness centrality of the key residues increases with upon addition of each component of the jasmonate receptor complex, by comparing systems 1 (COI1+ASK1), 2 (COI1+ASK1+1,5-InsP_8_), 3 (COI1+ASK1+1,5-InsP_8_+JA-Ile), and 6 (COI1+ASK1+JAZ1+1,5-InsP_8_+JA-Ile). While the closeness centrality of the key residues is smaller in system 4 (COI1+ASK1+JAZ1+JA-Ile) and 5 (COI1+ASK1+JAZ1+1,5-InsP_8_) than that in system 6 (COI1+ASK1+JAZ1+1,5-InsP_8_+JA-Ile). This implies that the information flows more rapidly with the help of 1,5-InsP_8_, JA-Ile, and JAZ1 in the COI1 solenoid.

**Table 3:** Summary of the shortest path betweenness and closeness centrality of selected residues in the six simulation systems.

**Residues**	**System 1**		**System 2**		**System 3**		**System 4**		**System 5**		**System 6**	
	**COI1**+**ASK1**		**COI1**+**ASK1**+**1,5-InsP**_**8**_		**COI1**+**ASK1**+**1,5-InsP**_**8**_+**JA-Ile**		**COI1**+**ASK1**+**JAZ1**+**JA-Ile**		**COI1**+**ASK1**+**JAZ1**+**1,5-InsP**_**8**_		**COI1**+**ASK1**+**JAZ1**+**1,5-InsP**_**8**_+**JA-Ile**	
	**Betweenness**	**Closeness**	**Betweenness**	**Closeness**	**Betweenness**	**Closeness**	**Betweenness**	**Closeness**	**Betweenness**	**Closeness**	**Betweenness**	**Closeness**
Met88	0.0778	0.1057	0.0029	0.1268	0.0044	0.1581	0.0816	0.1362	0.0112	0.1545	0.0028	0.1623
His118	0.0430	0.1103	0.0136	0.1527	0.0427	0.1634	0.0537	0.1288	0.0264	0.1624	0.0204	0.1643
Arg120	0.0083	0.1028	0.0098	0.1469	0.0049	0.1541	0.0091	0.1209	0.0105	0.1551	0.0069	0.1573
Arg121	0.0987	0.1079	0.0029	0.1452	0.0033	0.1536	0.0250	0.1311	0.0147	0.1546	0.0030	0.1568
Arg346	0.0462	0.0996	0.0225	0.1483	0.0901	0.1606	0.0066	0.1218	0.0173	0.1556	0.0164	0.1578
Tyr382	0.0758	0.1039	0.0155	0.1471	0.0209	0.1622	0.0121	0.1344	0.0161	0.1561	0.0079	0.1635
Arg409	0.1261	0.1084	0.0323	0.1484	0.0131	0.1586	0.0034	0.1327	0.0304	0.1570	0.0151	0.1635
Trp467	0.1284	0.1125	0.0182	0.1491	0.0103	0.1588	0.0349	0.1422	0.0130	0.1587	0.0116	0.1641
Lys492	0.0156	0.1069	0.0034	0.1470	0.0187	0.1558	0.0705	0.1412	0.0031	0.1556	0.0204	0.1613
1,5-InsP_8_	–	–	0.5270	0.1687	0.4795	0.1792	–	–	0.5765	0.1808	0.5105	0.1832
JA-Ile	–	–	–	–	0.2467	0.1683	0.4029	0.1506	–	–	0.2833	0.1728

### Predicted binding free energy for COI1—JA-Ile and COI1—JAZ1 complexes

To obtain the quantitativeestimation of the binding affinity of JA-Ile or JAZ1 with COI1, we performed binding free energy calculation by extracting 100 snapshots from the last 50 ns of the MD trajectories. The results were summarized in Table 4. The output energy terms include: the coulomb energy (ΔG_Coulomb_), the covalent binding energy (ΔG_Covalent_), the hydrogen-bonding energy (ΔG_Hbond_), the lipophilic energy (ΔG_Lipo_), the Van der Waals energy (ΔG_vdw_), the generalized Born electrostatic solvation energy ΔG_GB_, the Pi-pi packing energy (ΔG_Packing_), and the self-contact correction (ΔG_SelfCont_).

**Table 4:** The predicted binding free energy for different systems by Prime MM-GBSA method.

**System**	**Binding component**	Δ**Gbind**			Δ**G**_**Coulomb**_		Δ**G**_**Covalent**_		Δ**G**_**Hbond**_		Δ**G**_**Lipo**_		Δ**G**_**GB**_		Δ**G**_**vdw**_		Δ**G**_**Packing**_		Δ**G**_**SelfCont**_	
		**Average**	**STD**	***P*-value**	**Average**	**STD**	**Average**	**STD**	**Average**	**STD**	**Average**	**STD**	**Average**	**STD**	**Average**	**STD**	**Average**	**STD**	**Average**	**STD**
COI1+ASK1+1,5-InsP8+JA-Ile	COI1 JA-Ile	−109.83	3.98	8.5E−5^*^	−59.10	5.80	2.04	0.63	−2.82	0.22	−54.17	2.69	50.61	5.74	−46.39	2.09	–	–	–	–
COI1+ASK1+JAZ1+JA-Ile		−88.37	3.35	8.6E−93^*^	−43.40	5.37	1.81	1.03	−7.72	0.24	−49.57	1.61	49.74	4.76	−39.23	2.11	–	–	–	–
COI1+ASK1+JAZ1+1,5-InsP8 +JA-Ile		−110.77	4.46		−49.12	4.93	2.76	1.19	−3.07	0.25	−52.89	2.38	35.27	5.12	−43.72	2.58	–	–	–	–
COI1+ASK1+JAZ1+1,5-InsP8	COI1JAZ1	−188.66	5.72	1.1E−58^*^	−297.76	4.81	15.53	7.14	−18.08	4.02	−66.42	5.20	279.98	3.05	−102.30	5.82	0.42	2.10	0.03	0.67
COI1+ASK1+JAZ1+JA–Ile		−195.53	3.98	1.9E−42^*^	−284.20	4.25	4.86	7.56	−25.72	4.01	−65.59	7.49	286.86	3.47	−111.16	7.37	0.92	1.46	0.34	0.57
COI1+ASK1+JAZ1+1,5-InsP8 +JA-Ile		−207.04	4.94		−306.58	4.05	12.92	6.00	−19.48	4.33	−67.61	6.51	277.65	3.12	−104.83	8.32	0.50	1.20	0.39	0.26

* *Indicates significant difference at P < 0.05*.

The free energy components showed in Table 4 suggest that Δ*G*_coulomb_, ΔG_Lipo_, and Δ*G*_vdw_ are the majority of the favorable contributions for the binding of JA-Ile and JAZ1, whereas the polar solvation energies (Δ*G*_GB_) and ΔG_Covalent_ generate the unfavorable contributions. The predicted binding free energy for COI1—JA-Ile is −109.83 ± 3.98, −88.37 ± 3.35, and −110.77 ± 4.46 kcal/mol in system COI1+ASK1+1,5-InsP_8_+JA-Ile, COI1+ASK1+JAZ1+JA-Ile, and COI1+ASK1+JAZ1+1,5-InsP_8_+JA-Ile, respectively. The predicted binding free energy for COI1—JAZ1 is −188.66 ± 5.72, −195.53 ± 3.98, and −207.04 ± 4.94 kcal/mol in system COI1+ASK1+JAZ1+1,5-InsP_8_, COI1+ASK1+JAZ1+JA-Ile, and COI1+ASK1+JAZ1+1,5-InsP_8_+JA-Ile, respectively.

Based on the calculated binding free energy, the binding between COI1 and JA-Ile or JAZ1 is stronger in system with 1,5-InsP_8_ than those systems without 1,5-InsP_8_. The statistical analysis affirmed that the differences are significant (*P* < 0.05). The results indicate that the binding of InsP_8_ with COI1 promotes the binding between COI1 and JA-Ile or JAZ1.

## Discussion

The jasmonate receptor, a multi-component complex, consists of ASK1, COI1, inositol phosphate, and JAZ1. COI1, as a multi-component signaling hub, shares a conserved 3D structure with the auxin receptor TIR1. InsP plays important roles in hormone perception. How InsP binds with COI1 and the structural variations in COI1 that occur upon binding with InsP, JA-Ile, and JAZ are not well understood. Thus, a computational study was performed to elucidate the binding mechanism between the jasmonate receptor and InsPs.

Since there is no published crystal structure available for the InsP bound form of COI1, the different forms of InsPs were docked into the solenoid of COI1 in this study. The results suggest that 1,5-InsP_8_ binds to COI1 with the second lowest binding free energy and reasonable binding mode. 1,5-InsP_8_ forms hydrogen bond interaction with Lys79, Lys81, Arg85, His118, Arg120, Arg121, Arg346, Arg409, Lys492, from COI1 and Arg206 from JAZ1, which is also observed in Laha's work (Laha et al., 2015, 2016). Besides, it forms hydrogen bond interaction with Lys144 and Lys147 of COI1, which is not observed in Laha's work. It is not observed that 1,5-InsP_8_ forms hydrogen bond interaction with Arg440 of COI1 in our model, which is different from Laha's work. Residues His118, Arg346, and Lys492 of COI1 are observed to coordinate several phosphorus atoms of 1,5-InsP_8_, which is also reported in Laha's work (Laha et al., 2015). While Tyr382 of COI1 doesn't interact with 1,5-InsP_8_ in our model, which is different with Laha's model. Analysis of the detailed binding mode revealed that 1,5-InsP_8_ forms hydrogen bond interaction with His118, Arg346, Arg492 of COI1 and Arg206 of JAZ1, which is previously suggested to be important for the binding with InsP_8_ in the experimental work (Laha et al., 2015). Therefore, 1,5-InsP_8_ was chosen for further MD simulations.

MD simulations were conducted to elucidate the structural variations of COI1 that occur in response to binding with 1,5-InsP_8_, JA-Ile, and JAZ1. We found that the systems lacking JAZ1 [system 2 (COI1+ASK1+1,5-InsP_8_) and system 3 (COI1+ASK1+1,5-InsP_8_+JA-Ile)] exhibit higher variation than that in the other systems [system 4 (COI1+ASK1+JAZ1+JA-Ile), system 5 (COI1+ASK1+JAZ1+1,5-InsP_8_), and system 6 (COI1+ASK1+JAZ1+1,5-InsP_8_+JA-Ile)]. The JA-Ile also exhibits higher variation in system 3 than those in system 5 and system 6 (COI1+ASK1+JAZ1+1,5-InsP_8_+JA-Ile).

To further identify the flexible region of COI1, the RMSF of COI1 was plotted in each system. Through the RMSF plots (Figure 6), the flexible region of COI1 in response to binding with InsP, JA-Ile, and JAZ1 can be observed. Compared with the *apo* system and system 4 in the absence of InsP_8_, the α16-β14 loop, the α18-β16 loop, and the α14-β12 and the α17-β15 loop of COI1 in systems 2 and 3, system5 and system 6, respectively, exhibited high flexibility upon binding with InsP_8_. The β13-α16 and β16-α19 loops, the β17-α20 loop, and the β6-α8 loop in system 4, system 5, and system 6, respectively, showed higher flexibility upon binding with JAZ1. The flexible regions indicated their involvement in InsP_8_ or JAZ1 binding or entering the binding pocket in COI1. Due to JAZ1 is an intrinsically disordered protein (Chini et al., 2016), the flexible regions of COI1 found here would be helpful to suppose the binding mode of COI1 with other part of JAZ1, besides its conserved jas domain. Loop C exhibits high flexibility in all systems, which is based on the initial conformation by Rosetta and Modeler. Loop C forms tight interaction with JAZ1 in systems 3 to system 6, which supports the results of Sheard's study (Sheard et al., 2010), that loop C covers loop 2 and is involved in the binding with JAZ1. This conformation differs from that in the auxin receptor (Tan et al., 2007).

The interaction network in COI1 solenoid changes greatly upon the introduction of 1,5-InsP_8_, JA-Ile, and JAZ1. The detailed binding mode was analyzed for each system after MD simulation. We can find that some interactions disappear and some novel interactions form between 1,5-InsP_8_ and COI1. The conformation of 1,5-InsP_8_ also changes in those systems with or without JA-Ile and JAZ1 compared with system 6 (COI1+ASK1+JAZ1+1,5-InsP_8_+JA-Ile), implying that those components all affect the residue network in the COI1 solenoid. To further understand the residue interaction network, we performed the RIN analysis based on the structures extracted from the MD trajectories. The results indicated that 1,5-InsP_8_ and JA-Ile, with the highest betweenness values, act as the hub nodes and play crucial roles in the stabilizing the conformation of COI1 solenoid. The introduction of 1,5-InsP_8_ alters the residue interaction network in the COI1 solenoid, especially the interaction between Met88 and Arg121 or Lys147. Met88 then forms interaction of closest atom with JA-Ile and van der Waals interaction with JAZ1 (Figure 8F). Additionally, 1,5-InsP_8_ forms hydrogen bond interaction with Arg121, which explains why COI1 with a mutation of Met88 or Arg121 to alanine can't form a complex with JAZ1 even under the induction of coronatine (Sheard et al., 2010).

In view the importance of InsP_8_ in the network of COI1, it acts a necessary constituent and promotes the COI1-JAZ interaction when the levels of JA-Ile rise in response to a stimulus. In addition, based on the interaction between InsP_8_ and JAZ1, the doses of InsP_8_ would also modulate the conformation of COI1, which lead to its binding with different JAZ repressors in different conditions or responses. It was suggested that the detection of COI1—JAZ1, COI1—JAZ3, and COI1—JAZ9 required 60, 15, and 1.5 μM JA-Ile, respectively (Melotto et al., 2008; Chini et al., 2016). It can be conceived that InsP_8_ concentration would be another important factor in the specificity of COI1 among binding with different JAZ repressors.

## Conclusion

In the present study, the binding mechanism between InsP and COI1 was explored by molecular docking, molecular dynamics simulations, residue interaction network analysis and binding free energy calculation. The binding with 1,5-InsP_8_, JA-Ile, and JAZ1 makes the structure of COI1 more compact. In addition, the binding of 1,5-InsP_8_ with COI1 not only stabilizes the conformation of COI1 solenoid but also promotes the binding between JA-Ile or JAZ1 and COI1. Analysis the network parameters led to the identification of some hub nodes in this network, including Met88, His118, Arg120, Arg121, Arg346, Tyr382, Arg409, Trp467, and Lys492 of COI1. This study provides molecular basis on the recognition mechanism of each component of the jasmonate receptor complex. These results will facilitate the discovery and design of novel jasmonate signaling pathway modulators.

## Author contributions

JD conceived and supervised the experiments. MC performed MD simulations. MC and JD analyzed the data. MC, JD, and XY wrote the paper.

### Conflict of interest statement

The authors declare that the research was conducted in the absence of any commercial or financial relationships that could be construed as a potential conflict of interest.

## Supplementary material

Figure S1 Binding mode between different forms of inositol phosphates and the COI1-ASK1-JAZ1 complex predicted by molecular docking. The carbon atom and oxygen atoms of inositol phosphates and JA-Ile are colored in green and red, phosphorus atoms of inositol phosphates colored by orange, respectively. Hydrogens are omitted for clarity. **(A)** COI1+ASK1+JAZ1+JA-Ile+InsP_5_[3-OH]. **(B)** COI1+ASK1+JAZ1+JA-Ile+InsP_6_. **(C)** COI1+ASK1+JAZ1+JA-Ile+1,2-InsP_8_. **(D)** COI1+ASK1+JAZ1+JA-Ile+1,5-InsP_8_.
