# Supplementary figures and images for: The Binding Mechanism Between Inositol Phosphate (InsP) and the Jasmonate Receptor Complex: A Computational Study
Source: Front Plant Sci. 2018 Jul 18;9:963. doi: 10.3389/fpls.2018.00963 (PMC6058352; doi:10.3389/fpls.2018.00963)

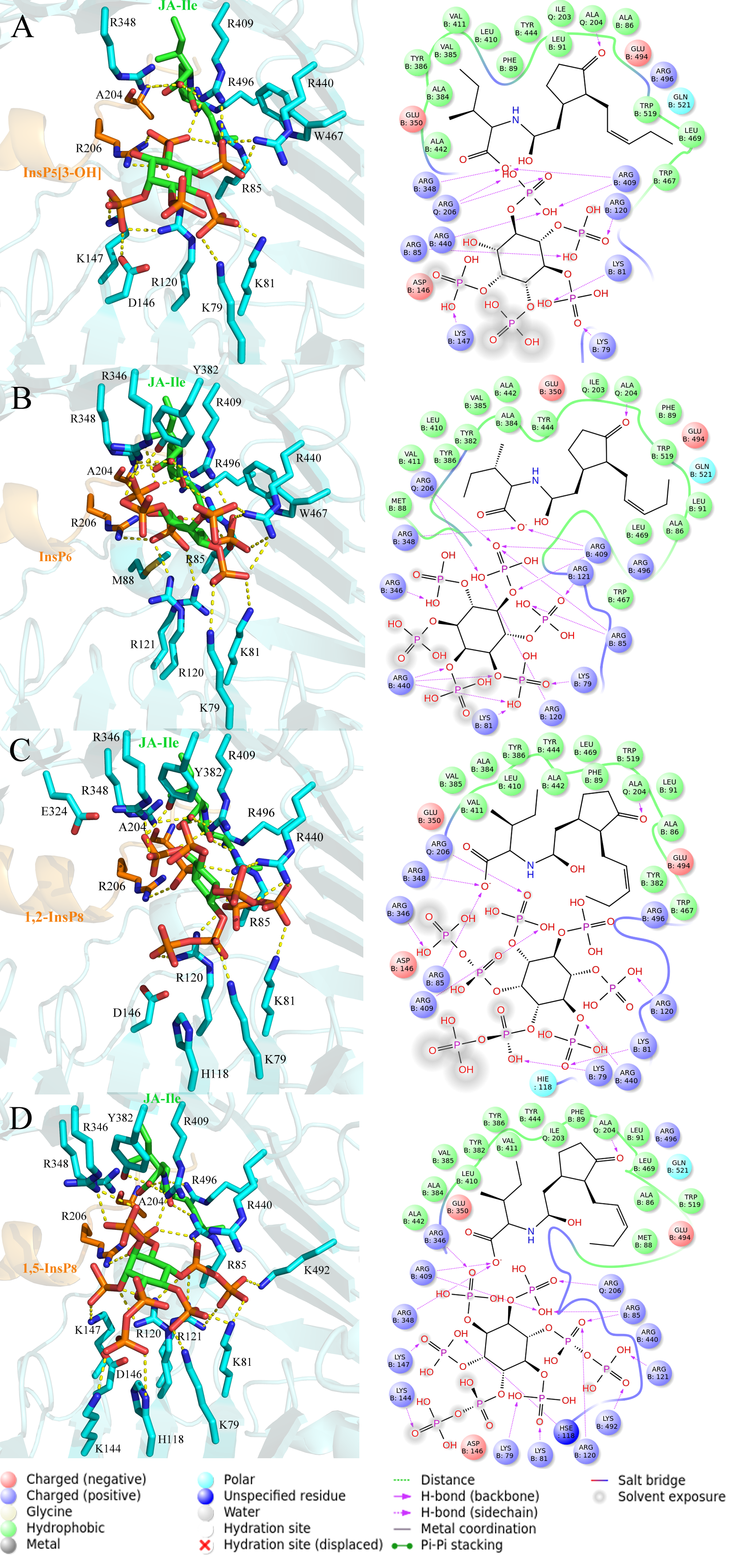

Supplement: Figure S1 — Binding mode between different forms of inositol phosphates and the COI1-ASK1-JAZ1 complex predicted by molecular docking. The carbon atom and oxygen atoms of inositol phosphates and JA-Ile are colored in green and red, phosphorus atoms of inositol phosphates colored by orange, respectively. Hydrogens are omitted for clarity. (A) COI1+ASK1+JAZ1+JA-Ile+InsP5[3-OH]. (B) COI1+ASK1+JAZ1+JA-Ile+InsP6. (C) COI1+ASK1+JAZ1+JA-Ile+1,2-InsP8. (D) COI1+ASK1+JAZ1+JA-Ile+1,5-InsP8. [file Image_1.TIF]
